# Update to CDC’s *U.S. Medical Eligibility Criteria for Contraceptive Use, 2016*: Revised Recommendations for the Use of Hormonal Contraception Among Women at High Risk for HIV Infection

**DOI:** 10.15585/mmwr.mm6637a6

**Published:** 2017-09-22

**Authors:** Naomi K. Tepper, Jamie W. Krashin, Kathryn M. Curtis, Shanna Cox, Maura K. Whiteman

**Affiliations:** 1 Div of Reproductive Health, National Center for Chronic Disease Prevention and Health Promotion, CDC.

CDC’s *U.S. Medical Eligibility Criteria for Contraceptive Use* (U.S. MEC) (first published in 2010 and updated in 2016) provides evidence-based guidance for the safe use of contraceptive methods among U.S. women with certain characteristics or medical conditions (*1*), and is adapted from global guidance from the World Health Organization (WHO) and kept up to date based on continual review of published literature (*2*).* CDC recently evaluated the evidence and the updated WHO guidance on the risk for human immunodeficiency virus (HIV) acquisition among women using hormonal contraception.^†^ After careful review, CDC adopted the updated WHO guidance for inclusion in the U.S. MEC guidance; this guidance states that the advantages of progestin-only injectable contraceptive use (including depot medroxyprogesterone acetate [DMPA]) by women at high risk for HIV infection outweigh the theoretical or proven risks (U.S. MEC category 2). The guidance also includes an accompanying updated clarification, which states that “there continues to be evidence of a possible increased risk of acquiring HIV among progestin-only injectable users. Uncertainty exists about whether this is due to methodological issues with the evidence or a real biological effect. In many settings, unintended pregnancies and/or pregnancy-related morbidity and mortality are common, and progestin-only injectables are among the few types of methods widely available. Women should not be denied the use of progestin-only injectables because of concerns about the possible increased risk. Women considering progestin-only injectables should be advised about these concerns, about the uncertainty over whether there is a causal relationship, and about how to minimize their risk of acquiring HIV.” Recommendations for other hormonal contraceptive methods (including combined hormonal methods, implants, and progestin-only pills) remain the same; there is no restriction for their use among women at high risk for HIV infection (U.S. MEC category 1).

## Background

Approximately half of pregnancies in the United States are unintended (*3*). Increasing access to and promoting correct and consistent use of contraception is a priority strategy to reduce unintended pregnancies. HIV infection continues to be a major public health issue in the United States.^§^ The vast majority of new infections among women are attributed to heterosexual contact.^¶^ HIV infection is associated with adverse pregnancy outcomes for both the mother and child, including increased morbidity during pregnancy and perinatal HIV transmission (*4*). Therefore, prevention of both unintended pregnancy and HIV acquisition is critical among women at high risk for HIV infection.

To date, recommendations for use of hormonal contraceptives among women at high risk for HIV infection have been U.S. MEC category 1 (safe for use without restriction) (Box). For women at high risk for HIV infection who use DMPA, a clarification was added in 2012 (*5*) and reaffirmed in 2016 (*1*), which described the inconsistent findings of studies examining a possible association between DMPA use and HIV acquisition and highlighted the importance of HIV preventive measures. CDC continually monitors published evidence as part of the process of keeping the U.S. MEC up to date. An update to U.S. MEC recommendations can be triggered by either identification of new evidence or an update to WHO global guidance. In March 2017, based on newly published studies (*6*), and after considering factors such as the balance of benefits and harms and ethical principles of ensuring informed contraceptive choice, WHO updated its recommendations on the safety of progestin-only injectable use among women at high risk for HIV infection from MEC category 1 to MEC category 2 (advantages of using the method generally outweigh the theoretical or proven risks).** WHO included a clarification that focuses on the possible increased risk of acquiring HIV with progestin-only injectable use, the limitations of the evidence, and the uncertainty about whether this represents a real biological effect. The clarification emphasizes that women should not be denied access to progestin-only injectables, but should be informed about these concerns and how to minimize risk for HIV acquisition. Because of newly published studies and the WHO update, CDC initiated a process to assess whether its guidance should be updated similarly for the U.S. context.

BOX **Categories for classifying hormonal contraceptives**

1 = A condition for which there is no restriction for the use of the contraceptive method.

2 = A condition for which the advantages of using the method generally outweigh the theoretical or proven risks.

3 = A condition for which the theoretical or proven risks usually outweigh the advantages of using the method.

4 = A condition that represents an unacceptable health risk if the contraceptive method is used.

## Methods

CDC considered several factors, including evidence on hormonal contraception use and risk for HIV acquisition, potential biologic mechanisms, and the context of contraception, unintended pregnancy, and HIV infection (e.g., incidence, demographics, and risk factors) in the United States. CDC invited seven participants from outside the agency and one participant from within the agency to serve as ad hoc reviewers of the evidence and the updated WHO recommendations (see “Participants”). The participants were selected based on their expertise in HIV infection or family planning. The participants joined one of two teleconferences with CDC staff members in May 2017 during which they reviewed the evidence, the updated WHO recommendations, and information on unintended pregnancy, contraceptive use, HIV infection, and maternal morbidity and mortality in the United States. The participants provided their individual input about 1) whether there has been a significant evolution in the evidence regarding hormonal contraception use and HIV acquisition, 2) how the updated evidence might influence clinical practice in the United States, and 3) how the updated WHO recommendations translate to clinical practice in the United States. After the teleconferences, CDC developed the recommendations in this report, taking into consideration the individual perspectives provided by the participants.

## Rationale and Evidence

A systematic review of published evidence regarding the use of hormonal contraception and the risk for HIV acquisition was published in 2016 (*6*). The systematic review included primary research studies (randomized trials or observational studies) identified in PubMed or Embase databases through January 2016. Included studies reported on incident HIV infection among women using hormonal contraception (injectables, oral contraceptives, implants, patches, rings, or hormonal intrauterine devices) compared with incidence among women using nonhormonal or no contraception. Studies were excluded if they did not report a risk estimate for hormonal contraception and HIV acquisition, were cross-sectional studies, only assessed emergency contraception, or were conference abstracts. Study quality was evaluated using a framework developed for previous reviews on this topic, and assessment focused on 31 studies considered to be “informative but with important limitations” (*6*). These higher quality studies included adjustment for condom use and had clear measurement of exposure to hormonal contraceptives. Among 11 studies evaluating the association between oral contraceptive use and HIV acquisition, 10 found no statistically significant association between oral contraceptive use and risk for HIV acquisition, while one reported a marginally significantly increased association. Evidence from 13 studies evaluating the association between progestin-only injectable contraceptives and risk for HIV acquisition suggested a possible increased risk (adjusted hazard ratio = 1.4 [95% confidence interval = 1.2–1.6] among 10 studies specifically examining DMPA), but findings were inconsistent across studies and limited by methodologic concerns. Two studies of levonorgestrel implants and one study of progestin-only pills did not suggest increased risk for HIV acquisition.

In an additional study published after the systematic review and identified using the same search strategy, women in South Africa were randomized to receive either copper intrauterine devices or progestin-only injectable contraceptives (*7*). The study found no increased risk for HIV acquisition with progestin-only injectable contraceptive use. This is the only randomized trial examining this issue; however, the study was subject to many limitations including a small sample size (approximately 20 women in each group acquired HIV), high loss to follow-up (25%), self-report of final HIV status for one third of participants, and no information on contraceptive switching or discontinuation (*7*).

Animal and laboratory data suggest a range of possible biologic mechanisms for an association between hormonal contraceptive use and HIV acquisition, potentially related to the progestin component, including hormonally mediated changes in the vaginal epithelium and alterations in local and systemic immune responses (*8*,*9*). However, the relevance of these observations to clinical outcomes in women is unclear (*8*,*9*).

Whereas overall use of DMPA in the United States was low (4.5%) among current contraceptive users during 2011–2013, use was higher among black women (10%), those aged 15–24 years (8.5%), those who had income <150% of the federal poverty level (7.3%), and women who had less than a high school education (10.1%).^††^ Although the rate of unintended pregnancy is declining, 45% of pregnancies in the United States were unintended in 2011, with higher percentages among women aged 15–19 years (75%) and black women (64%) (*3*). Pregnancy-related mortality in the United States also differs significantly by race, with approximately a threefold higher risk among black compared with white women (*10*). In 2015, an estimated 7,400 new HIV infections occurred among U.S. women, with higher rates among minorities.^§§^^,^^¶¶^ Although use of DMPA and risk for HIV are lower in the United States than in many areas globally, the prevalence of DMPA use in the United States is higher among subgroups of women who have characteristics associated with increased risk for HIV infection, unintended pregnancy, and pregnancy-related complications.

## Recommendations for the Use of Hormonal Contraceptives in Women at High Risk for HIV

For implants, progestin-only pills, and combined hormonal contraceptives, U.S. MEC recommendations remain the same as those in the U.S. MEC 2016: these methods can be used without restriction among women at high risk for HIV infection (U.S. MEC category 1) (Table). For DMPA, CDC adopted the updated WHO recommendation that the advantages of DMPA use outweigh the theoretical or proven risks among women at high risk for HIV infection (U.S. MEC category 2). In accordance with WHO, CDC updated the clarification for DMPA, which highlights that there continues to be evidence of a possible increased risk for HIV acquisition among women using progestin-only injectable contraceptives, but it is not clear whether this is a real biological effect or due to methodological issues with the studies; that U.S. women should not be denied DMPA because of concerns about this possible increased risk; and that women considering DMPA should be advised about these concerns, as well as about HIV prevention measures. The complete U.S. MEC guidance, including recommendations about use of copper and levonorgestrel-releasing intrauterine devices by women at high risk for HIV (which were not reviewed for this update), are available at https://www.cdc.gov/reproductivehealth/contraception/usmec.htm.

**TABLE:** Recommendations for contraceptive use by women who are at high risk for human immunodeficiency virus (HIV) infection

Condition	Category				Clarifications/Evidence
	Implants	DMPA	POP	CHCs	
**High risk for HIV**	1	2	1	1	**Clarification (DMPA):** There continues to be evidence of a possible increased risk of acquiring HIV among progestin-only injectable users. Uncertainty exists about whether this is due to methodological issues with the evidence or a real biological effect. In many settings, unintended pregnancies and/or pregnancy-related morbidity and mortality are common, and progestin-only injectables are among the few types of methods widely available. Women should not be denied the use of progestin-only injectables because of concerns about the possible increased risk. Women considering progestin-only injectables should be advised about these concerns, about the uncertainty over whether there is a causal relationship, and about how to minimize their risk of acquiring HIV. **Evidence (Implants, DMPA, POP):** Evidence from 13 observational studies of DMPA, NET-EN or nonspecified progestin-only injectables, which were considered to be “informative but with important limitations,” continues to show some association between use of progestin-only injectables and risk of HIV acquisition, but it remains unclear whether this results from a causal relationship or methodological limitations.* One additional randomized pilot feasibility trial, published subsequently to the systematic review, found no statistically significant difference in risk of HIV acquisition between progestin-only injectable users (DMPA or NET-EN) and copper IUD users; this study had several limitations including lack of power to assess differences in HIV acquisition rates, and problems with ascertainment of hormonal contraception exposure and HIV acquisition outcomes.^†^ Two small studies assessing levonorgestrel implants, which were considered to be “informative but with important limitations,” did not suggest an elevated risk, although the risk estimates were imprecise.* One study reported no association between use of progestin-only pills and HIV acquisition.* **Evidence (CHCs)**: Eleven studies, deemed “informative but with important limitations,” assessed the use of OCs. Ten of these studies found no statistically significant association between use of OCs and HIV acquisition, while one study reported a marginally significant increased risk. No studies of patch, ring or combined injectable contraception were identified.*

**Abbreviations:** CHC = combined hormonal contraceptive; DMPA = depot medroxyprogesterone acetate; HIV = human immunodeficiency virus; IUD = intrauterine device; NET-EN = norethisterone enanthate; OC = oral contraceptive; POP = progestin-only pills.

* Polis CB, Curtis KM, Hannaford PC, Phillips SJ, Chipato T, Kiarie JN, et al. An updated systematic review of epidemiological evidence on hormonal contraceptive methods and HIV acquisition in women. AIDS 2016;30:2665–83. http://journals.lww.com/aidsonline/fulltext/2016/11130/An_updated_systematic_review_of_epidemiological.13.aspx.

^†^ Hofmeyr GJ, Singata-Madliki M, Lawrie TA, Bergel E, Temmerman M. Effects of injectable progestogen contraception versus the copper intrauterine device on HIV acquisition: sub-study of a pragmatic randomised controlled trial. J Fam Plann Reprod Health Care 2017;43:175–80. http://jfprhc.bmj.com/content/familyplanning/early/2017/04/05/jfprhc-2016-101607.full.pdf.

## Discussion

CDC adopted the updated WHO guidance for inclusion in the U.S. MEC guidance. Although the U.S. context differs from the global context in a number of ways (e.g., generally lower DMPA use, lower HIV incidence, greater access to a range of contraceptive methods, and lower risks for maternal morbidity and mortality), issues related to possible risks and the need for counseling are relevant across settings. Current data continue to suggest a potential increased risk for HIV acquisition with DMPA use, although significant limitations in data quality remain. Despite the previous U.S. MEC clarification stating that women at high risk for HIV should be counseled about risks and benefits of DMPA, some of the experts consulted by CDC expressed concern that this is not occurring in clinical practice in the United States or globally, and an updated recommendation might encourage providers to counsel women on risks, benefits, and alternatives to DMPA. CDC does not intend for a change from an MEC category 1 to MEC category 2 to result in decreased access to DMPA. CDC’s guidance is intended for health care professionals, and CDC is committed to working with professional organizations and other stakeholders to assist in interpretation and implementation of these recommendations in all clinical settings. Evaluating changes in practice associated with updated recommendations might be useful for assessing implementation by providers, administrators, and organizations caring for women at high risk for HIV infection. CDC anticipates that these recommendations will lead to improvements in provider training and patient education materials reflecting the risks and benefits of DMPA use. DMPA continues to be a safe, effective, and practical contraceptive method for many women.

Access to the full range of safe and effective Food and Drug Administration–approved contraceptive methods is essential for women at high risk for HIV infection to avoid unintended pregnancy. For women at high risk for HIV infection who wish to use DMPA, the advantages outweigh the theoretical or proven risks, and women should not be denied access to this method. Evidence of a possible increased risk for HIV acquisition among users of progestin-only injectable contraceptives (including DMPA) remains inconclusive. HIV infection prevention measures should be strongly encouraged among all women at risk for HIV acquisition, including limiting numbers of sexual partners, correct and consistent use of condoms, and consideration of preexposure and postexposure prophylaxis.***

## Footnotes

*
http://www.who.int/reproductivehealth/publications/family_planning/MEC-5/en .

† http://www.who.int/reproductivehealth/publications/family_planning/HC-and-HIV-2017/en/.

§ https://www.cdc.gov/hiv/library/reports/hiv-surveillance.html.

¶ Approximately 6,400 out of 7,400 HIV diagnoses in 2015 (https://www.cdc.gov/hiv/pdf/library/reports/surveillance/cdc-hiv-surveillance-report-2015-vol-27.pdf).

**
http://apps.who.int/iris/bitstream/10665/254662/1/WHO-RHR-17.04-eng.pdf?ua=1 .

†† https://www.cdc.gov/nchs/data/nhsr/nhsr086.pdf.

§§
https://www.cdc.gov/nchhstp/atlas/index.htm .

¶¶ https://www.cdc.gov/hiv/basics/statistics.html.

***
https://www.cdc.gov/hiv/basics/prevention.html .
